# Investigation of Photoprotective, Anti-Inflammatory, Antioxidant Capacities and LC–ESI–MS Phenolic Profile of *Astragalus gombiformis* Pomel

**DOI:** 10.3390/foods10081937

**Published:** 2021-08-20

**Authors:** Sabrina Lekmine, Samira Boussekine, Salah Akkal, Antonio Ignacio Martín-García, Ali Boumegoura, Kenza Kadi, Hanene Djeghim, Nawal Mekersi, Samira Bendjedid, Chawki Bensouici, Gema Nieto

**Affiliations:** 1 Laboratory of Bioactive Molecules and Applications, Larbi Tébessi University, Tébessa 12000, Algeria; sabrina.lekmine@univ-tebessa.dz (S.L.); samira.boussekine@univ-tebessa.dz (S.B.); 2 Valorization of Natural Resources, Bioactive Molecules and Biological Analysis Unit, Department of Chemistry, University of Mentouri Constantine 1, Constantine 25000, Algeria; salah.akkal@umc.edu.dz; 3 Estación Experimental del Zaidín (CSIC), ProfesorAlbareda 1, 18008 Granada, Spain; ignacio.martin@eez.csic.es; 4 Biotechnology Research Center (C.R.Bt), Ali Mendjeli, Nouvelle Ville, UV 03 BP E73, Constantine 25000, Algeria; a.boumegoura@crbt.dz (A.B.); h.djeghim@crbt.dz (H.D.); c.bensouici@crbt.dz (C.B.); 5 Biotechnology, Water, Environment and Health Laboratory, Abbes Laghrour University, Khenchela 40000, Algeria; kadi.kenza@univ-khenchela.dz (K.K.); mekersinawal@univ-khenchela.dz (N.M.); 6 Research Laboratory of Functional and Evolutionary Ecology, Department of Biology, Faculty of Natural Sciences and Life, Chadli Bendjedid University, El Tarf 36000, Algeria; samiraphyto@gmail.com; 7 Department of Food Technology, Food Science and Nutrition, Faculty of Veterinary Sciences, Regional Campus of International Excellence “Campus Mare Nostrum”, Espinardo, 30071 Murcia, Spain

**Keywords:** *Astragalus gombiformis* Pomel, photoprotective effect, anti-inflammatory activity, antioxidative activity, cytotoxicity, phenolic compounds, LC–ESI–MS

## Abstract

Plant-derived compounds have recently been gaining popularity as skincare factors due to their ability to absorb ultraviolet radiations and their anti-inflammatory, and antioxidant properties. In this light, this work aimed to evaluate in vitro the pharmacological activities of the butanolic extract prepared from the aerial parts of *Astragalus gombiformis* Pomel, an endemic species to southern Algeria. The sun protection factor was used to assess the photoprotective effect (SPF), the protein denaturation method to determine the anti-inflammatory activity, and brine shrimp nauplii and OxHLIA assay, respectively, to assess the cytotoxicity and antioxidant capacity of *A. gombiformis*. In addition, LC–ESI–MS analysis was employed for the characterization of the phenolic constituents of *A. gombiformis*. The results showed that *A. gombiformis* had high capacity for absorbing UV radiations with an SPF of 37.78 ± 0.85 and significant anti-inflammatory activity with a percentage inhibition of 75.38% which is close to that of diclofenac and ketoprofen. In addition, *A. gombiformis* was found to have effective cytotoxicity against *Artemia nauplii* with a DC50 value of about 44.7 µg/mL, but a weak hemolytic effect against human erythrocytes. LC–ESI–MS results detected the presence of 17 phenolic compounds with a predominance of cirsiliol, silymarin, quercitrin (quercetin-3-*O*-rhamnoside), and kaempferol. Taken together, these results suggest that *A. gombiformis* extract could be used as a skincare agent in cosmetic formulations, providing excellent antioxidant and anti-inflammatory protection, allowing the treatment of skin conditions, as well as a pharmaceutical agent with multidimensional applications.

## 1. Introduction

The skin is the most regenerative organ in the body, representing a barrier with important roles, such as the regulation of water balance, thermogenesis, and permeability; hence, it is considered a natural defense mechanism against microbial infections [1,2]. However, this protective barrier can be continuously damaged by several environmental factors and biological insults. For example, excessive exposure to solar radiation without protection allows UV radiation to penetrate the skin—in particular, UV-A and UV-B, which stimulate the generation of reactive oxygen species (ROS) such as (O_2_), (OH), and (H_2_O_2_) [3].

Excessive ROS production may contribute to the ineffectiveness of natural antioxidant systems by disrupting cell homeostasis, leading to oxidative stress, and damaging proteins, lipids, and nucleic acids [4]. It is widely accepted that oxidative stress is a major factor responsible for the initiation or progression of several illnesses, such as skin elasticity, wrinkles, alterations in elastic fibers and collagen color, oxidative hemolysis, inflammatory diseases, and skin cancer caused by genetic mutations [5]. In this context, many scientists are interested in medicinal plants as substitutional natural sources of antioxidants and anti-inflammatory and photoprotective compounds with few side effects [6].

The study of medicinal plants reveals locally important species that are often useful for discovering new bioactive products [7,8]. In line with this, several studies have been carried out onthe characteristics of several species of *Astragalus*, the largest genus of the *Fabaceae* family that includes more than 3000 species and represents one of the most important legumes widely used in the food and pharmaceutical industries due to itsstrong antioxidant capacity, which is attributed to the presence of many bioactive secondary metabolites [9]. This genus is distributed in Mediterranean climatic regions in Europe and North Africa [10], where fifteen species have been found in the Sahara Desert of Algeria, as well asten species endemic to Morocco and Tunisia [11,12].

Various species of *Astragalus* are used in traditional and modern medicine due to their biological effects, such as antioxidant, antibacterial, and antiviral [13]. In Turkey, for instance, the roots of *Astragalus* species are widely used asa remedy for leukemia and for wound healing purposes [14]. However, in China, *A. mongholicus* Bunge and *A. membranaceus* Bunge are among the most popular medicinal plants commonly used as an adjunct in cancer chemotherapy [15,16]. Several products of *Astragalus*, such as gum tragacanth, are used in the preparation of pharmaceuticals and as thickening agents in food products [17]. Many bioactive chemicals from this genus have been shown to be useful in the treatment of cancer cells, [18] such as saponins isolated from *A. corniculatus M. Bieb*, which exhibited a strong antineoplastic effect against myeloid tumors in hamsters [19].

Several *Astragalus* species are widespread in North Africa, including *A. gombiformis* Pomel, which grows in arid regions. This species is traditionally used in cases of snake and scorpion bites, probably due to the presence of active products that act against scorpion envenomation [20]. This species also contains several pharmacologically active compounds, such as phenolics and polysaccharides with immunostimulatory effects [21].

Considering its richness in bioactive compounds, the presence has been reported of a considerable concentration of flavonoids, alkaloids, proanthocyanidols, tannins, and saponosides in the *A. gombiformis* plant growing in Tunisia [21]. The essential oils and the MeOH extract of this Tunisian species were found to be rich in complex mixtures of natural compounds, such as phytol, 6,10,14-trimethyl-2-pentadecanone, 4-terpineol, gamma-terpinene, 7-methyl quercetin 3-*O*-α-l-rhamnopyranosyl-(1→2)-β-D-galactopyranoside (2), and 7-methyl quercetin 3-*O*-α-L-rhamnopyranosyl-(1→2)-[6-*O*-(3-hydroxy-3-methylglutaryl)-β-D-galactopyranoside [22,23].

Given the great interest in new bioactive molecules for functional ingredients in different food, cosmetic, and pharmaceutical industries, we undertook the present work as a primary biological and chemical search of dry land forage legume *Astragalus gombiformis* Pomel, used to feed local cattle in arid and semi-arid regions of southern Algeria. To date, no studies have investigated the anti-inflammatory, photoprotective, and cytotoxic activities of *A. gombiformis*. To this end, we performed this study with the following aims: (1) to assess the photoprotective, anti-inflammatory, antioxidant, and cytotoxic activities of *A. gombiformis* aerial part extract; and (2) to characterize the chemical constitution of phenolic compounds of the whole plant extract using LC–ESI–MS.

## 2. Materials and Methods

### 2.1. Reagents and Standards

#### 2.1.1. Chemicals and Reagents for Extraction and Spectrophotometric Determinations of Biochemical Activities

The solvents utilized were obtained from PROLAB, MERK EUROLAB. Folin-Ciocalteu, sodium carbonate (Na_2_CO_3_), aluminum nitrate (Al(NO_3_)), potassium acetate (CH_3_CO_2_K), sulfuric acid (H_2_SO_4_), acetic acid (C_2_H_4_O_2_), dimethyl sulfoxide (DMSO), vanillin, and quillajasaponaria of highest purity (≥99.0%) were purchased from Sigma-Aldrich Chemie (St. Louis, MO, USA) and FlukaChemie GmbH (Buchs, Switzerland).

#### 2.1.2. Reagents for LC/MS Analysis

The following standards were obtained from Sigma Chemical Co (St. Louis, MO, USA) at the highest purity available, as shown in Table 1: quinic acid, gallic acid, protocatchuic acid, catechin (+), caffeic acid, syringic acid, 1,3-di-*O*-caffeoyquinic acid, epicatechin, *p*-coumaric acid, rutin, *trans*-ferulic acid, hyperoside (quercetin-3-*O*galactoside), luteolin-7-*O*-glucoside, 3,4-di-*O*-caffeoyquinic acid, naringin, rosmarinic acid, 4,5-di-*O*-caffeoyquinic acid, quercetin (quercetin-3-*O*-rhamonoside), apigenin-7-*O*-glucoside, *O*-coumaric acid, Salvianolic acid, kaempferol, quercetin, *trans* cinnamic, silymarin, naringenin, apigenin, luteolin, cirsiliol, cirsilineol, acacetin.

### 2.2. Plant Collection and Extraction

*A. gombiformis* plants were collected from their natural habitats in the Saharan region (EL Oued-Algeria). The aerial parts were cleaned, air-dried, grounded, and stored in paper bags until use. Extraction was performed according to the method outlined in Bensouici et al., 2019 [24] and Lekmine et al., 2020 [25]. In all, 200 g of *A. gombiformis* was extracted with 2 L of ethanol–water (70:30 *v*/*v*) for 24 h. The residue was suspended in water and extracted using petroleum ether and butanol (only the butanolic fraction was used in further experiments).

### 2.3. Determination of Total Bioactive Compounds

#### 2.3.1. Total Phenolic Content (TPC)

TPC was estimated spectrophotometrically using the Folin–Ciocalteu method [26,27]. Briefly, 20μL of the extract was added to 100 μL of Folin–Ciocalteu reagent (diluted ten-fold *v/v*) and 75 μL (75 g/L) of sodium carbonate. After 2 h incubation in darkness, the absorbance was read at 740 nm. The results were expressed as microgram of gallic acid equivalents per milligram of extract (μg GAE/mg extract).

#### 2.3.2. Total Flavonoid Content (TFC)

TFC was measured using the colorimetric technique of Topçu et al., 2007 [28]. In total, 50 μL of extract was added to 10 μL of aluminum nitrate (10% *w*/*v*), 10 μL of potassium acetate (1 M), and 130 μL of methanol. After 40 min incubation, the absorbance was read spectrophotometrically at 415 nm. Quercetin was used as the reference compound, and the results were expressed as microgram quercetin equivalents per milligram of extract (μg QE/mg extract).

#### 2.3.3. Total Saponin Content (TSC)

TSC was determined via the vanillin–sulfuric acid method of Hiai et al. (1976) [29]. Briefly, 50 µL of butanolic extract was mixed with vanillin (8%, *w/v* 0.5 mL) and sulfuric acid (72%, *w/v* 5 mL). After incubation at 60 °C for 10 min and cooling in an ice water bath for 15 min, the absorbance was read at 538 nm. Quillaja saponin (*Quillaja saponaria*) is used as a reference compound [30], and the total saponin content is expressed as quillaja saponin equivalents (µg QSE/mg extract).

### 2.4. Liquid Chromatography–Electrospray Ionization–Tandem Mass Spectrometry (LC–ESI–MS) Analysis

The phenolic profile was determined viaLC–ESI–MS analysis using a Shimadzu UFLC XR system (Kyoto, Japan), equipped with a SIL-20AXR auto-sampler, a CTO-20 AC column oven, a LC-20ADXR binary pump, and a quadripole 2020 detector system.For analysis, an Aquasil C18 column (Thermo Electron, Dreieich, Germany) (150 mm × 3 mm, 3 μm) preceded by an Aquasil C18 guard column (10 mm × 3 mm, 3 μm, Thermo Electron) was used. The mobile phase was composed of A (0.2% acetic acid in 5% MeOH and 95% H_2_O, *v*/*v*) and B (0.2% acetic acid in 50% CAN and 50% H_2_O, *v*/*v*) with a linear gradient elution: 0–45 min, 10–100% B; 45–55 min, 100% B. Re-equilibrate duration was 5 min between individual runs. The injection volume was 20 μL, the flow rate of the mobile phase was 0.4 mL/min, and the temperature of the column was maintained at 40 °C. Spectra were monitored in selected-ion-monitoring (SIM) mode and processed using Shimadzu LabSolutions LC–MS software. The mass spectrometer was operated in negative ion mode with a capillary voltage of −3.5 V, a nebulizing gas flow of 1.5 L/min, a dry gas flow rate of 12 L/min, a dissolving line (DL) temperature of 250 °C, a block source temperature of 400 °C, a voltage detector of 1.2 V, and the full scan spectra from 50 to 2000 *m*/*z*. Phenolic compound identification was achieved throughcomparison with retention times of standard compounds [31].

### 2.5. Photoprotective Activity

The photoprotective activity of *A. gombiformis* extract against UV damage was assessed throughin vitrodetermination of the sun protection factor (SPF) [32]. The extract was dissolved in absolute methanol, and absorbance was measured at 290–320 nm and 5 nm intervals, using a UV spectrophotometer (Shimadzu UV-1700, Japan). The SPF value was calculated using the following formula:

(1) $$\mathrm{SPF}\;=\mathrm{CF}\overset{320}{\underset{290}{\sum }}\mathrm{EE}\left(\lambda \right)I\left(\lambda \right)\;\mathrm{Abs}\left(\lambda \right)$$

where EE(λ) is the erythemal effect spectrum, I(λ) is the solar intensity spectrum, Abs(λ) is the absorbance, and CF is the correction factor (CF = 10).The values of EE(λ) × I(λ) are constant [33].

### 2.6. Anti-Inflammatory Activity

In vitro anti-inflammatory activity was tested via the protein denaturation method using bovine serum albumin (BSA) as described by Karthik et al. (2013) [34]. To 0.5 mL of different concentrations of the extract or reference compounds (ketoprofen and diclofenac), 0.5 mL of BSA (0.2% *w*/*v*) in Tris-HCl buffer (pH 6.8) was added. Tightly closed tubs were incubated in the oven at 37 °C for 15 min and then heated in a water bath at 70 °C for 5 min. The absorbance of turbidity was taken at 660 nm. The percentage inhibition was calculatedas follows: %I = (1 − At/Ac) × 100; where At is the absorbance of test sample and Ac is the absorbance of control.

### 2.7. Oxidative Hemolysis Inhibition Assay (OxHLIA)

Antioxidant activity was evaluated using an oxidative hemolysis inhibition assay (OxHLIA) [35,36].First, in tubes containing heparin, five milliliters of blood was taken from a healthy person and used to prepare a suspension of erythrocytes. The collected blood was centrifuged at 1500× *g* for 3 min. Plasma was removed, and the pellet was washed many times with asterile phosphate buffer saline (PBS; pH7.2). Then, the erythrocytes were resuspended by gentle shaking in a normal saline suspension (0.5%). A total of 500 μL of the different concentrations of the test extract (125, 250, 500, and 1000 μg/mL) prepared in PBS was mixed with 500 μL of the cell suspension. The resultingmixtures were incubated at 37 °C for 30 min and centrifuged at 1500× *g* for 5 min. The absorbance was measured spectrophotometrically at 540 nm. Finally, the following formula was used to determine the percentage of hemolysis:

[(Ac − At)/Ac] ×100(2)

where Ac is the absorbance of control and Atis the absorbance of test (in the presence ofalkaloids).

%H = [(At − An)/(Ac − An)] × 100(3)

AE: the absorbance of the extract;
AP: the absorbance of the positive control (phosphate buffer saline);
AN: the absorbance of the negative control (distilled water).

### 2.8. Brine Shrimp Lethality Test (BST)

The *A. gombiformis* extract was tested against nauplii of brine shrimp (*Artemia salina*) [37]. Briefly, the vial was filled with artificial seawater to which 200 mg of *Artemia salina* eggs was added. After 48 h incubation at 30 °C. In an illuminated incubator, using a Pasteur pipette, phototropic *Artemia nauplii* were collected. In all, 100 μL of a solution of dimethyl sulfoxide (DMSO)–seawater (4%) containing various concentrations (0.5; 1; 2; 4 mg/mL) was transferred to tubes containing 4.9 mL of filtered seawater and 10 *Artemia* larvae. The surviving shrimp were counted after 24 h, and the percentage of mortality (deaths) was determined.

### 2.9. Statistical Analysis

All measurements were carried out in triplicate, and data arereported as means ± SD. The results were subjectedto one-way analysis of variance (ANOVA) using PRISM (GraphPad software 5.0, San Diego, CA, USA followed by a Tukey HSD test (α = 0.05).

## 3. Results

### 3.1. Bioactive Compounds

**Figure 1:**
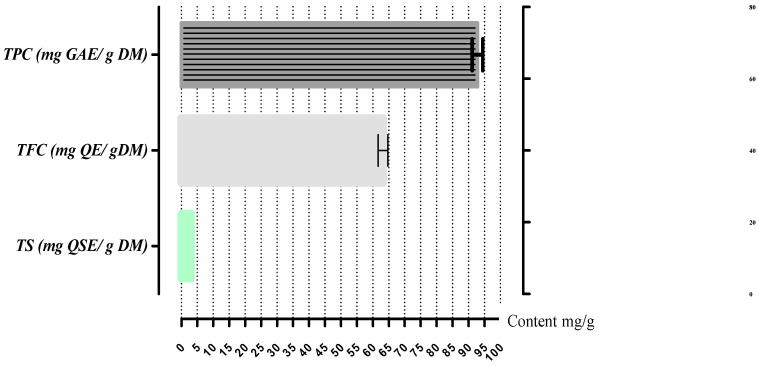
Total bioactive compounds of *A. gombiformis* extract. Bars represent standard deviation.

The *A. gombiformis* extract presented a TPC of 92.8 ± 1.69 mg GAE/g DM, mainly due to the TFC (63.2 ± 1.56 mg QE/g DM), which was composed oflow amounts of TSC (2.97 ± 00 mg QSE/g DM), as shown in Figure 1.

### 3.2. Identification and Quantification of Phenolic and Flavonoid Compounds

**Figure 2:**
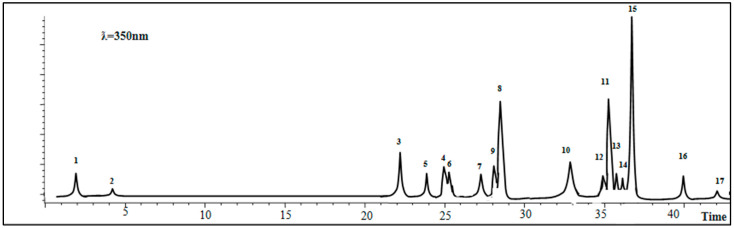
Total ion chromatograms (TIC) of the active extract of *A. gombiformis* obtained by HPLC–ESI–MS analysis in negative ionization mode. 1: Quinic acid; 2: Gallic acid; 3: *P*-coumaric acid; 4: Rutin; 5: *Trans*-ferulic acid; 6: Hyperoside (quercetin-3-*O*-galactoside); 7: Rosmarinic acid; 8: Quercitrin (quercetin-3-*O*-rhamnoside); 9: Apigenin-7-*O*-glucoside; 10: Kaempferol; 11: Silymarin; 12: Naringenin; 13: Apigenin; 14: Luteolin; 15: Cirsiliol; 16: Cirsilineol; 17: Acacetin.

The results of LC–ESI–MS analysis of the *A. gombiformis* extract are shown in Figure 2 and summarized in Table 1.

**Table 1:** Phytochemicals identified in the extract of *A. gombiformis*.

	Compound	% Purity	Rt	([M-H]-)	Concentration (mg/100 g DM)	RSD Curve Calibration	(R^2^)	Linear Range (μg/mL)	LOD (μg/mL)	LOQ (μg/mL)
1	Quinic acid	98	2.017	191.00	6.16 ± 0.28	14.320	0.9952	0.05–7.5	0.616	1.867
2	Gallic acid	97.4	4.317	169.00	1.16 ± 0.75	10.548	0.9999	0.05–7.6	0.102	0.308
3	*p*-coumaric acid	98	22.283	163.00	8.99 ± 0.78	5.560	0.9981	0.05–7.5	0.337	1.022
4	Rutin	98	25.256	609.00	4.12 ± 0.19	11.809	0.9991	0.05–20.0	0.172	0.521
5	*trans*-ferulic acid	99	24.550	193.00	2.34 ± 0.73	5.231	0.9982	0.05–7.5	0.624	1.890
6	Hyperoside (quercetin-3-*O*-galactoside)	98	25.829	463.00	2.15 ± 0.56	10.851	0.9964	0.05–20.0	0.115	0.349
7	Rosmarinic acid	98	27.876	359.00	4.45 ± 0.48	8.618	0.9995	0.05–15.0	0.115	0.454
8	Quercitrin (quercetin-3-*O*-rhamnoside)	91.4	28.050	447.00	14.01 ± 0.20	10.970	0.9996	0.05–5.0	0.171	0.520
9	Apigenin-7-*O*-glucoside	98	28.028	431.00	1.87 ± 0.39	12.817	0.9989	0.05–2.0	0.821	2.489
10	Kaempferol	97	33.350	285.00	10.05 ± 0.90	12.466	0.9985	0.05–5.0	0.148	0.450
11	Silymarin	>95	35.398	481.00	14.76 ± 0.65	13.218	0.9952	0.05–20.0	0.051	0.154
12	Naringenin	95	35.083	271.00	1.29 ± 0.25	10.058	0.9970	0.05–2.0	0.115	0.349
13	Apigenin	>95	35.717	269.00	1.41 ± 0.16	11.067	0.9981	0.05–1.0	0.068	0.206
14	Luteolin	97	36.283	285.00	1.99 ± 0.09	12.376	0.9973	0.05–5.0	0.516	1.565
15	Cirsiliol	95	36.975	329.00	44.46 ± 0.36	12.911	0.9982	0.05–5.0	0.030	0.090
16	Cirsilineol	95	40.139	343.00	1.68 ± 0.19	6.743	0.9977	0.05–2.0	0.181	0.548
17	Acacetin	≥99	42.117	283.00	0.92 ± 0.11	20.134	0.9987	0.10–7.5	0.085	0.258

Data are presented as mean ± SD of three parallel measurements (*n* = 3); Rt: retention time; LOD: limits of detection; LOQ: limits of quantification.

The LC–ESI–MS analysis detected the presence of 17 phenolic compounds, of which 4 were detected as main compounds: cirsiliol (44.46 mg/100 g DM), silymarin (14.76 mg/100 g DM), quercitrin (quercetin-3-*O*-rhamnoside) (14.01 mg/100 g DM), andkaempferol (10.05 mg/100 g DM).On the other hand, six other compounds had moderate concentrationsranging between 2 and 8 mg/100 g DM including *p*-coumaric acid (8.99 mg/100 g DM), quinic acid (6.16 mg/100 g DM), rosmarinic acid (4.45 mg/100 g DM), rutin(4.12 mg/100 g DM), *trans*-ferulic acid (2.34 mg/100 g DM), and hyperoside (quercetin-3-*O*-galactoside) (2.15 mg/100 g DM). Moreover, gallic acid, cirsilineol, Apigenin-7-*O*-glucoside, naringenin, apigenin, luteolin, and acacetin were found as traces with the lowest values (<2 mg/100 g DM).

### 3.3. Photoprotective Activity

As shown in Table 2, the *A. gombiformis* extract showed a high SPF (37.78 ± 0.85), with high absorbance values that ranged between 4.143 and 3.698 at *λ* = 290–320 nm.

### 3.4. Anti-Inflammatory Activity

**Figure 3:**
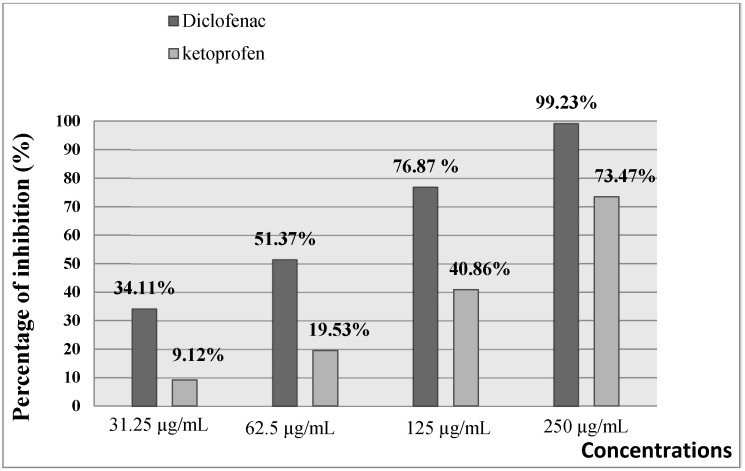
In vitro anti-inflammatory effect of diclofenac (Standard 1) and ketoprofen (Standard 2).

**Figure 4:**
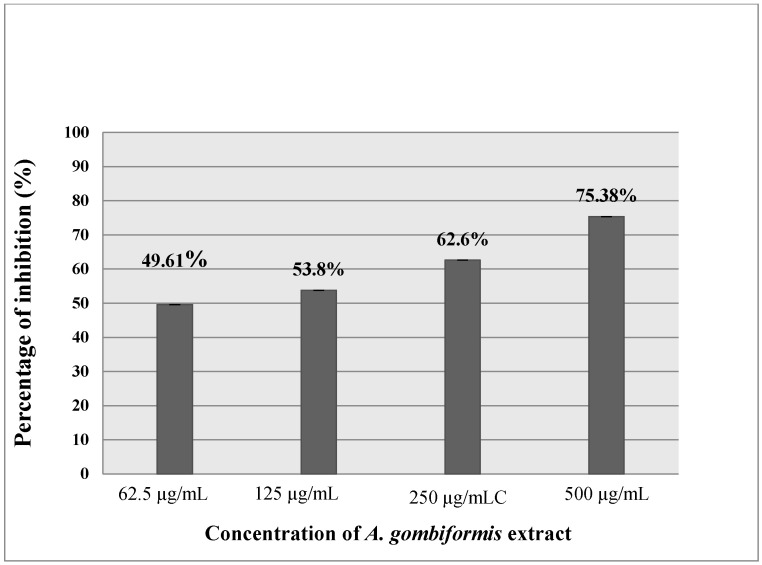
In vitro anti-inflammatory effect of *A. gombiformis* extract.

As presented in Figure 3 and Figure 4, the extract of *A. gombiformis*, as compared to the reference molecules (diclofenac and ketoprofen), showed a concentration-dependent inhibitory activity against the protein denaturation induced by the high temperature. At 500 µg/mL, a significant anti-inflammatory effect was obtained with a percentage of inhibition of 75.38%, which was close to the inhibition scores of diclofenac (99.23%) and ketoprofen (73.47%) at 250 µg/mL. The EC50 of the extract tested (69.42 ± 0.02 µg/mL) was higher compared to the ketoprofen standard (165.83 ± 0.103µg/mL) and very close to the EC50 of the diclofenac standard (63.5 ± 0.02 µg/mL) (Table 3).

### 3.5. OxHLIA Assay

**Figure 5:**
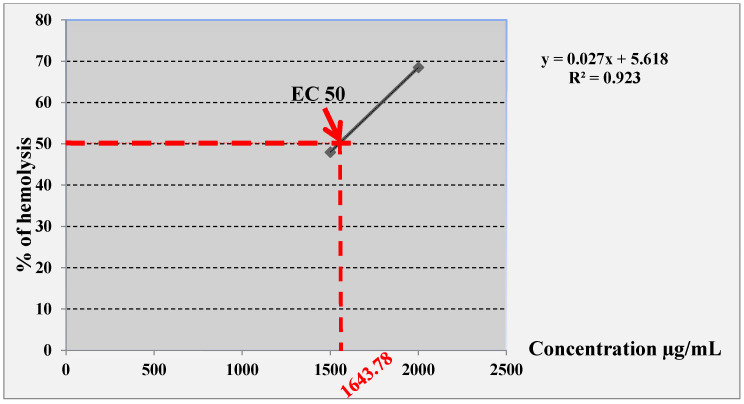
Linear equation of the curve to determine the EC50 of *A. gombiformis* butanolic extract.

The results of the butanolic extract obtained from *A. gombiformis* are represented in Figure 5. The butanolic fraction exhibited a low hemolytic effect toward human erythrocytes. However, the hemolytic activity of this extract was dose-dependent, i.e., it increased with the increase in extract concentration. The EC50 was estimated at 1643.78 µg/mL, which was calculated according to the linear equation: y = 0.027x + 5.618 (R^2^ = 0.923).

### 3.6. Brine Shrimp Lethality Bioassay (BSLB)

The results of mortality are shown in Table 4. No mortality was detected within the control group treated with DMSO, while the *A. gombiformis* extract exhibited strong cytotoxicity with a DC50 of 44.7 ± 1.76 μg/mL.

## 4. Discussion

*Astragalus gombiformis* is a medicinal plant species endemic to Algeria. With an interesting chemical composition, it is commonly used in traditional medicine in North Africa for the treatment of a wide range of illnesses. In the present work, we evaluated the photoprotective, anti-inflammatory, and antioxidant activities of the butanolic fraction of this interesting plant species.

Phenolic acids are one of the most numerous classes of secondary metabolites, withgreat diversity in structure and properties [38]. Based on this study, phytochemical screening of butanolic extract revealed that *A. gombiformis* has considerable concentrations of phenolic compounds, flavonoids, and saponins. The TPC obtained in this study (92.8 ± 1.69 mg GAE/g DM) was higher compared to the that of the methanolic extract (9.19± 0.27 mg GAE/g DM) of the aerial parts of *A. gombiformis* growing in Tunisia [39]. The same trend was registered by Sevil and Onur (2019) [40] for methanolic extract of *Astragalus argaeus* Boiss from Turkey (10.4 ± 0.3 mg GAE/g DM). The TPC results of this study are higher than those obtained by Bronislava et al. (2018) [41] from *A. glycyphyllos* extract of leaves and flowers (25.99 and 23.71 mg GAE/g DM).

The TFC results of this study are higher compared to the TFC of *A.argaeus* in the variation of methanolic extract (5.88 ± 0.1 mg QE/g DM) [40]. Moreover, TFC is relatively higher compared to flavonoid content in the extract of *A. glycyphyllos* in leaves (21 mg RE/g) and flowers (16.71 mg RE/g) [41]. The high phenolic contents in *Astragalus gombiformis* increase itsnutritional and therapeutic values. The TS content islow compared to that of phenolic compounds (2.97 mg *Quillaja*/g DM). Several factors have been reported to influence the TPC of plants, including geographical origin [42], environmental and ecological conditions [43], variety and degree of maturation [44], and finally extraction conditions [45,46].

In order to confirm the above results, a complete liquid chromatography–electrospray ionization–tandem mass spectrometry (LC–ESI–MS) analysis was optimized and validated to quantify 31 phytochemical fingerprint compounds (phenolic and flavonoids compounds) [31].The results showed the presence of cirsiliol, silymarin, quercitrin(quercetin-3-*O*-rhamonosid), kaempferol, *p*-coumaric acid, quinic, rosmarinic acid, rutin, *trans*-ferulic acid, hyperoside (quercetin-3-*O*-galactoside), gallic acid, cirsilineol, apigenin-7-*O*-glucoside, naringenin, apigenin, luteolin, and acacetin.These results indicated a relatively larger number of compounds compared to that determined in Lekmine et al. (2020) [9], where the presence of just 12 compounds, with low concentrations in stems, flowers, leaves, pods, and seeds of *A. gombiformis*, was detected. The emergence of new compounds from the total plant extract, whichwere not previously recorded by LC–MS of the five separate organs, could be explained by the synergistic effect between the compounds present in each organ. This interaction may cause the synthesis of new derived molecules demonstrated in the present analysis. Moreover, this finding can also be explained by the presence of these new molecules in each organ, but with low concentrations which are not detectable by LC–MS. Thus, the combination of the whole plant resulted in an increase in concentrations and therefore in their emergence in the LC–ESI–MS analysis.

Furthermore, the previous phenolic compounds cited above were detected in *A. schizopterus* methanol extract [47]. Similarly, the major phenolic compounds obtained, such as kaempferol, quercetin, rutin, and rosmarinic, were detected using two HPLC methods in different *Astragalus* species [48,49]. Moreover, the characterization of phenolic compounds in *Astragalus quisqualis* and *Astragalus kabadianus* was well documented [50,51]. Eight phenolic compounds were isolated and purified from *Astragalus taipaishanensis*, and their structures were elucidated using ESI–MS, HR-ESI–MS, 1D-NMR, and 2D-NMR in the form of 7,2′-dihydroxy-3′, 4′-dimethoxy isoflavane, formononetin, isoliquiritigenin, quercetin, kaempferol, ononin, *p*-hydroxybenzoic acid, and vanillic acid [52]. The variation of this chemical constitution is related to several factors, such as ecological and climate conditions, genotypes, and environmental stress within the geographical positions of the plant material tested [53]. According to Mollaeiet al. (2020) [54], environmental factors had a significant effect on the essential oil content and antioxidant activity of *Mentha pulegium* L. Additionally, Mehalaine and Chenchouni (2020, 2021) [55,56] extensively explained how edaphic variables and climatic factors influence the accumulation of essential oils in wild plants in North Africa. Based on the literature and the present results, we can consider *A. gombiformis* an important source of phenolic compounds with potential for biomedical applications due to its secondary metabolism that is dependent on local ecological conditions.

The deleterious effects of exposure to ultraviolet (UV) radiation on skin have become more apparent. Numerous sunscreen and skincare products have therefore been developed to help to reduce the occurrence of sunburn, photoaging, as well as skin carcinogenesis. In this vein, this study has stimulated research on using new natural sources of effective skin-protecting compounds. The photoprotective activity of *A. gombiformis* has not been previously investigated. Therefore, the data presented in this study represent an original contribution to the literature. An excellent capacity to absorb UV radiation was registered by *A. gombiformis* extract (SPF = 37.78 ± 0.85). Skin-protecting products having SPF values greater than 30 are considered to be effective UV-radiation filters [57].This finding could be explained by the presence of rosmarinic acid that has been considered a photoprotective agent against UV and other ionizing radiations [58]. It has a high protective effect against the unfavorable influence of methylparaben and propylparaben on collagen in human skin fibroblasts [59]. This attenuates cell damage induced by UV-B radiation via enhancing the antioxidant defense system in human HaCaT cells [60].Therefore, extract *of A. gombiformis* can be employed as a sun protection product in sunscreen formulations to protect the skin from sunburn. Our findings are consistent with what is known about many crude extracts prepared from various *Astragalus* spp., which are wellknown for their pharmaceutical applications. According to Curnow and Owen. 2016 [61], *Astragalus membranaceus* and *Althea officinalis* are considered natural sources for UV-protecting dermatological formulations. We may also conclude that this photoprotective capacity may be due to the climate conditions of the Saharan region of *A. gombiformis*, as daily exposure to the sun leads to the production of more bioactive compounds to protect the plant from UV damage. Several studies have revealed thatthe significant abilities of UV absorption are associated with the chemical constituents, especially flavonoids and phenolics [62]. Based on the results of this work, there is a strong correlation between total phenolic contents and the photoprotective activity of *A. gombiformis* extract. According to the literature, these compounds are considered excellent sun filters with significant photoprotective effects [63,64]. Moreover, the presence of cyclic and aromatic hydrocarbons offers the ability to absorb ultraviolet light with wavelengths ranging between 240–285 nm and 300–550 nm [65].

The anti-inflammatory activity of *A. gombiformis* extract was carried out using theprotein denaturation method. The principal mechanism of this denaturation consists of the alteration of electrostatic, hydrophobic, hydrogen, and disulfide bonds that maintain the three-dimensional structure of proteins [66,67]. After denaturation, most proteins lose their biological functions, causing the production of autoantigens inducing several autoimmune dysfunctions, including rheumatic and inflammatory diseases. Therefore, agents which inhibit protein denaturation are considered to be effective anti-arthritis and anti-inflammatory drugs [68]. The results of in vitro anti-inflammatory activity showed an excellent ability of this plant extract to maintain the three-dimensional structure of proteins. These findings are in agreement with the results obtained for diclofenac, whichis used as a standard by Mouffouk et al. (2020) [69] and possesses an important anti-inflammatory effect (inhibition = 86.72%) at the same concentration as that in which *A. gombiformis* extract was tested (500 ug/mL). A good correlation was obtained between phenolic compounds and flavonoids identified previously via the LC–ESI–MS technique and anti-inflammatory activity. Nevertheless, this anti-inflammatory capacity exhibited by this plant extract could be attributed to the presence of the main bioactive molecules that have not been detected viaLC–MS analysis, such as fatty acids, carotenoids, and steroids. This hypothesis can be confirmed through the in vitro anti-inflammatory activity of cycloartane type saponins from *Astragalus* species that was investigated by Nalbantsoy et al. (2012) [70].

The findingsregarding OxHLIA activity are related to the chemical composition of the butanolic extract obtained from *A. gombiformis*, such as saponins. These compounds are widely known for their ability to alter membranes by inducing pore formation or the permeabilization of the erythrocyte membrane due to their amphiphilic properties [71]. The presence of other metabolites, such as alkaloids or phenolic compounds, can also induce hemolysis [72].

According to the literature, several studies have shown that saponins can induce the lyses of erythrocytes [71,73], such as iridoids, which constitute the main cause of the hemolysis observed when using the *n*-BuOH and EtOAc extracts of *S. stellata.* In addition, iridoid glycosides cause hemolytic anemia and a decrease in red blood cells and hemoglobin. Accordingly, based on the results we obtained through phytochemistry screening, we may conclude that this ability is due to the presence of saponins [49], which could be isolated and utilizedas afood ingredient flavor enhancer and anti-yeast agentaccording to Golmohammadi (2013) [74]. Indeed, some species of *Astragalus* have been used as a source of many stabilizers and commercial thickening agents, such as Tragacanth (E413). From a nutritional point of view, *Astragalus* seeds are an important source of protein, carbohydrates, polyunsaturated fatty acids (PUFA), microelements, and vitamins, which are the most substantial nutrients for human beings [11].

Brine shrimp bioassay was used to elucidate the possible toxicity of bioactive compounds in extracts which are generally toxic in high doses [75]. According to Mouffouk et al. (2020) [76], the secondary metabolites present in *Noneavesicaria* extract induced direct damage on membrane integrity by causing cell lysis. Therefore, these compounds may likely have cytotoxic effects, something that may also apply to *A. gombiformis*. However, previous studies have shown that plant extract lethality against brine shrimp nauplii with a value of DC50 below 100 μg/mL is reasonably correlated with cytotoxic and antitumor properties and may constitute potential antitumor and anticancer agents [77]. Therefore, the cytotoxicity observed in this study could be related to the chemical profile of the tested plant and its metabolite content, such as saponins, whichwere detected previously through phytochemical screening and which are well known as antitumor agents with cytotoxic effects and antiproliferative potential [78]. Despite the low cytotoxic effect of the plant against *Artemia Salina*, it remains a plant with low side effects whose *Astragalus* genus is mostly used as fodder crop for both livestock and wild animals in dry regions as well as in industrial foods as a tea flavoring agent, coffee substitute, and source of natural gum, and in cosmetics and pharmaceutical medicines [11].

## 5. Conclusions

The present study reported, for the first time, an extensive evaluation of the biological activities of *Astragalus gombiformis* Pomel, an endemic species from Algeria. The phytochemical screening of butanolic extract indicated the presence of various types of secondary metabolites with interesting pharmacological activities. This extract had moderate antioxidant activity but a great ability to absorb UV radiation, whereas *A. gombiformis* extract showed good efficiency in terms of thermallyinduced protein denaturation in a dose-dependent manner. Moreover, the plant extract showed high potential therapeutic application, as evidenced through a cytotoxic test. Economically, it is advised to use the whole plant to offer greater returns compared to each organ separately, because of the considerable concentration of the phenolic compounds identified viaLC–MS analysis in the whole plant. Taken together, the high contents of bioactive compounds play more significant roles as novel raw materials for functional foods and promising chemical additives in the food and pharmaceutical industry. Based on the above findings, the *A. gombiformis* plant can be considered an important source of bioactive components in therapeutic medicine, as well as in cosmetics applications as a photoprotective factor with an anti-inflammatory effect and antioxidant response induced by UV radiation.

## Figures and Tables

**Table 2:** Values of sun protection factor of the *Astragalus gombiformis* extract.

λ (nm)	EE(λ) × I(λ) (Norms)	*A. gombiformis* Extract.	
		Absorbance	SPF
290	0.0150	4.143 ± 0.01	0.621 ± 0.00
295	0.0817	3.862 ± 0.02	3.155 ± 0.02
300	0.2874	3.843 ± 0.00	11.046 ± 0.01
305	0.2780	3.682 ± 0.26	12.069 ± 0.85
310	0.1864	3.802 ± 0.00	7.087 ± 0.00
315	0.0837	3.733 ± 0.00	3.132 ± 0.00
320	0.0180	3.698 ± 0.00	0.665 ± 0.00
Total	1		37.78 ± 0.85

EE(λ): erythemal effect spectrum; I(λ): solar intensity spectrum; SPF: sun protection factor.

**Table 3:** In vitro anti-inflammatory effect of *A. gombiformis* extract and reference compounds.EC50 values are expressed as means ± SD of three replicates. The same subscript letters are not significantly different following the Tukey’s honestly significant difference post hoc test at *p* < 0.05.

Extract/Reference Compound	EC50 (µg/mL)
Diclofenac	63.5 ± 0.02 ^a^
Ketoprofen	165.83 ± 0.103 ^c^
*A. gombiformis* extract	69.42 ± 0.02 ^b^

**Table 4:** Brine shrimp lethality bioassay of butanolic fraction extracted from *A. gombiformis* compared to K_2_Cr_2_O_7_ used as standard.

*A. gombiformis* Butanolic Fraction μg/mL	*A. gombiformis* % of Mortality	K_2_Cr_2_O_7_ % of Mortality
10	16.7 ± 5.77	0 ± 0.00
20	26.7 ± 5.77	50 ± 0.00
40	43.3 ± 5.77	80 ± 10.00
80	86.7 ± 5.77	100 ± 0.00
DC50 (μg/mL)	44.7 ± 1.76	20.6
